# Mendelian randomization study supports positive bidirectional causal relationships between genetically predicted insomnia symptom and liability to benign prostatic hyperplasia

**DOI:** 10.1186/s12894-024-01474-z

**Published:** 2024-04-20

**Authors:** Nannan Li, Ke Yang, Liang Deng, Youjie Zeng, Si Cao, Dong Chen

**Affiliations:** 1 The First Hospital of Changsha, Changsha, 410005 China; 2 The Affiliated Changsha Hospital of Xiangya School of Medicine, Central South University, Changsha, 410008 China; 3 Department of Anesthesiology, Third Xiangya Hospital, Central South University, Changsha, Hunan 410013 China; 4 Clinical Research Center for Reproduction and Genetics in Hunan Province, Reproductive and Genetic Hospital of CITIC-XIANGYA, Changsha, Hunan 410205 China

**Keywords:** Mendelian randomization, Incidence risk, Insomnia, Prostatic Hyperplasia, Risk factors

## Abstract

**Background:**

Sleep quality may be related to benign prostatic hyperplasia (BPH), however causal associations have not been established. This study aimed to evaluate causal relationships between six sleep traits ([i] day time napping, [ii] daytime sleepiness, [iii] insomnia, [iv] long sleep duration, [v] short sleep duration, and [vi] sleep duration per hour) and BPH through a bidirectional Mendelian randomization (MR) study.

**Methods:**

Genome-wide association summary statistics of sleep traits and BPH were downloaded from public databases. Inverse variance weighting (IVW) was used as the main approach for causal inference. For causal estimates identified by IVW, various sensitivity analyses were performed to assess the reliability of the results: (i) four additional MR methods to complement IVW; (ii) Cochran’s Q test to assess heterogeneity; (iii) MR-Egger intercept test and MR-PRESSO global test to assess horizontal pleiotropy; and (iv) leave-one-out method to assess stability.

**Results:**

Forward MR analyses indicated that genetically predicted insomnia symptom significantly increased BPH risk (OR = 1.267, 95% CI: 1.003–1.601, *P* = 0.048), while reverse MR analyses identified that genetically predicted liability to BPH significantly increased the incidence of insomnia (OR = 1.026, 95% CI: 1.000-1.052, *P* = 0.048). In a replicate MR analysis based on summary statistics including exclusively male participants, the finding of increased risk of BPH due to genetically predicted insomnia symptom was further validated (OR = 1.488, 95% CI: 1.096–2.022, *P* = 0.011). No further causal links were identified. In addition, sensitivity tests demonstrated the reliability of the MR results.

**Conclusion:**

This study identified that a higher prevalence of genetically predicted insomnia symptoms may significantly increase the risk of BPH, while genetically predicted liability to BPH may in turn increase the incidence of insomnia symptom. Therefore, improving sleep quality and reducing the risk of insomnia could be a crucial approach for the prevention of BPH.

**Supplementary Information:**

The online version contains supplementary material available at 10.1186/s12894-024-01474-z.

## Introduction

Benign prostatic hyperplasia (BPH), often referred to as prostate enlargement, is a non-cancerous condition characterized by the enlargement of the prostate gland [1]. Primarily observed in older males, this condition is linked with a variety of urinary symptoms, including difficulty initiating urination, weak urine stream, and frequent urination, particularly at night [1]. With the exacerbation of global population aging, the prevalence of BPH has caused substantial economic burdens. Despite thorough investigations, the pathophysiology of BPH remains incompletely understood. One prevailing theory underscores the pivotal role of age-induced alterations and disturbances in hormonal equilibrium as the primary contributors to its development [2]. BPH treatment includes meds (alpha blockers, 5-ARIs, combo therapy), surgeries (TURP, HoLEP, Aquablation), and lifestyle changes (diet, exercise, bladder training) [3]. While these approaches alleviate symptoms, concerns about side effects and the need for continual management persist, underscoring the necessity for novel preventative strategies.

Generally, the risk factors for BPH encompass approximately five categories, namely chronological aging, hereditary factors, hormonal influences, modifiable lifestyle factors, and inflammatory processes [4]. While individuals with BPH commonly encounter nocturia, insomnia, and other sleep-related issues [5], recent studies have shown that sleep quality can also influence the incidence of BPH [6, 7]. Notably, the prevalence of sleep disorders tends to be higher among the elderly, affecting 50–60% of this demographic [8]. Sleep assumes a critical role in nurturing the central nervous system as well as rejuvenating physical abilities [9]. Moreover, inadequate sleep is associated with disturbances in immune function, metabolic processes, and hormonal functions [10]. Nonetheless, findings from observational studies could not elucidate the causality between sleep and BPH.

Mendelian randomization (MR) analysis represents a novel statistical approach that utilizes genetic variants as instrumental variables (IVs) to infer causal relationships between exposures and outcomes [11]. Due to the random assignment of genetic variations at the time of embryonic development, which is minimally influenced by postnatal environments, MR analysis holds the advantage of reducing confounders and lowering the likelihood of reverse causality [11]. The accessibility of summary statistics of genome-wide association study (GWAS) has significantly streamlined MR studies, guaranteeing robust statistical analyses owing to substantial sample sizes. In situations where conducting randomized controlled trials (RCTs) is challenging or unethical, such as interventions related to sleep, MR studies serve as a valuable alternative. Here, a bidirectional MR study was performed to comprehensively assess the causal association between sleep traits and BPH, thereby providing evidence for the prevention of BPH from the perspective of improving sleep quality.

## Methods

### Study design

**Fig. 1:**
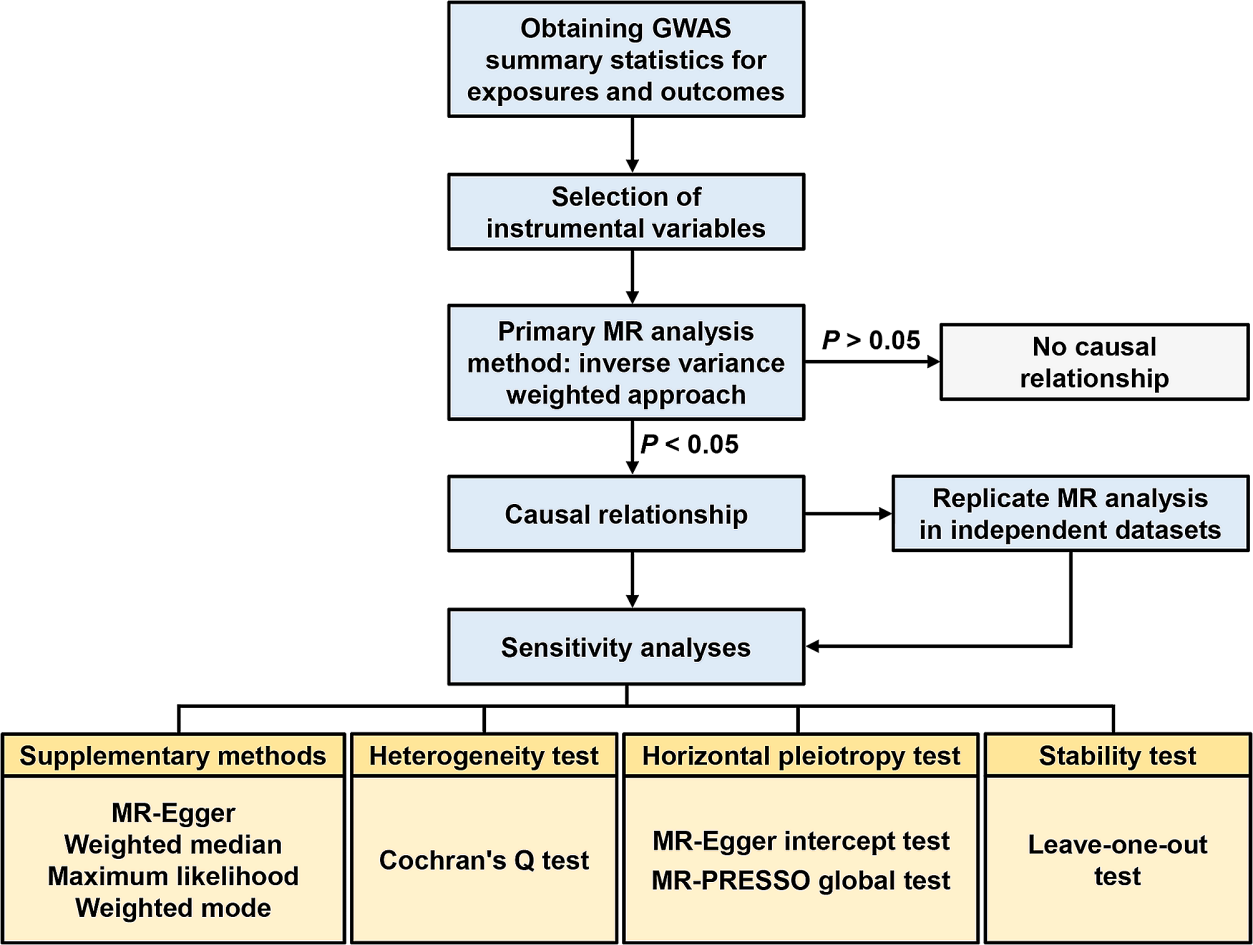
General flowchart of this MR study

Figure 1 exhibits the general flow of this study. Specifically, GWAS summary statistics for the six sleep traits ([i] day time napping, [ii] daytime sleepiness, [iii] insomnia, [iv] long sleep, [v] short sleep, and [vi] sleep duration per hour) and BPH were first obtained from publicly available databases. Subsequently, single-nucleotide polymorphisms (SNPs) related to each sleep trait were utilized as the IVs for the forward MR analysis, and SNPs related to BPH were utilized as the IVs for the reverse MR analysis. Then, the main approach, inverse variance weighted (IVW), was performed to assess causality. Furthermore, for the established causal associations, various sensitivity analyses were further implemented to evaluate the reliability of the results. Finally, we validated the main findings of this study by performing replicate MR analyses using GWAS summary statistics that included exclusively male participants.

### Obtaining GWAS summary statistics

Detailed information of all GWAS summary statistics is displayed in Supplementary Table S1. Summary statistics of the six sleep traits, including day time napping [12], daytime sleepiness [13], insomnia [14], long sleep [15], short sleep [15], and sleep duration per hour [15], were derived from GWAS studies conducted based on participants from the UK Biobank cohort. GWAS summary statistics for BPH were derived from the FinnGen R9 cohort, which consisted of 30,066 patients and 119,297 healthy controls [16]. Since sleep characteristics and BPH were from two separate cohorts, there was no overlap of participants, thus avoiding bias. Since this study was a secondary analysis based on public data, no extra ethical consent was required.

### Selection of IVs

IVs were identified in accordance with three core assumptions: First, IVs were significantly related to exposures. Second, IVs were independent of confounders. Third, IVs were not directly related to outcomes. SNPs associated with sleep traits were utilized for forward MR analysis, while SNPs related to BPH were used for reverse MR analysis. Specifically, first, SNPs related to exposure were screened from summary statistics (*P* < 5e-8). Next, SNPs related to potential confounders were excluded. Considering that educational attainment, smoking, and alcohol consumption may be associated with both insomnia and BPH, and the fact that they impact numerous other phenotypes [17–21], we included these three phenotypes as potential confounders in this study. Source of summary statistics for the confounders and the relevant SNPs are exhibited in Supplementary Tables S2–S3. Next, SNPs exhibiting linkage disequilibrium (r^2^ < 0.001 within 10,000 kb) were eliminated and those with the smallest *P*-values were retained. In addition, all SNPs were not significantly associated with outcome (*P* > 5e-8). Ultimately, the strength of each IVs was assessed using *F*-statistics, and only IVs with *F*-statistics exceeding 10 could mitigate the weak IV bias.

### Statistical analysis

IVW was the main MR approach to assess causality. Initially, the causal impact of the exposure on the outcome was evaluated using the Wald ratio method individually for each IV. Subsequently, a meta-analysis was conducted employing either fixed-effects or random-effects models [22]. The significance level was set at *P* < 0.05 to determine statistical significance. For causal relationships identified by IVW, a variety of sensitivity tests were performed to assess reliability. First, several additional MR methods, including MR-Egger [23], weighted median [24], maximum likelihood [25], and weighted mode [26], were employed for supplementing IVW. Subsequently, heterogeneity was calculated by Cochran’s Q test. Following this, both the MR-Egger intercept and MR-PRESSO global test were performed to assess horizontal pleiotropy. Finally, the leave-one-out test was performed to assess stability.

### Replicate MR analysis

We further validated the causal effect of insomnia on BPH with an independent external GWAS summary statistics. Watanabe et al.‘s recent large-scale GWAS meta-analysis comprehensively integrated GWAS data on insomnia phenotypes from the UK Biobank and 23andMe [27]. We obtained summary statistics for insomnia GWAS in male participants of the study, including 222,753 male cases and 993,280 male controls. Data on male-specific SNPs significantly associated with insomnia were obtained from the Supplementary Table of the original manuscript. The procedures for IV selection, MR analysis, and sensitivity testing were consistent with those described previously in the current study.

## Results

### **Identification of IVs for** forward and reverse MR analysis

The IVs screening process for the forward MR analysis is shown in Supplementary Table S4. In the forward MR analysis, 7 to 87 SNPs were used as IVs proxying six sleep traits (Supplementary Table S5). The IVs screening process for the reverse MR analysis is shown in Supplementary Table S6. In the reverse MR analysis, 34 to 40 IVs representing BPH were used to evaluate the causal impact of BPH on six sleep traits (Supplementary Table S7). *F*-statistics of all IVs were > 10.

### IVW approach identified bidirectional causality between Insomnia and BPH

**Fig. 2:**
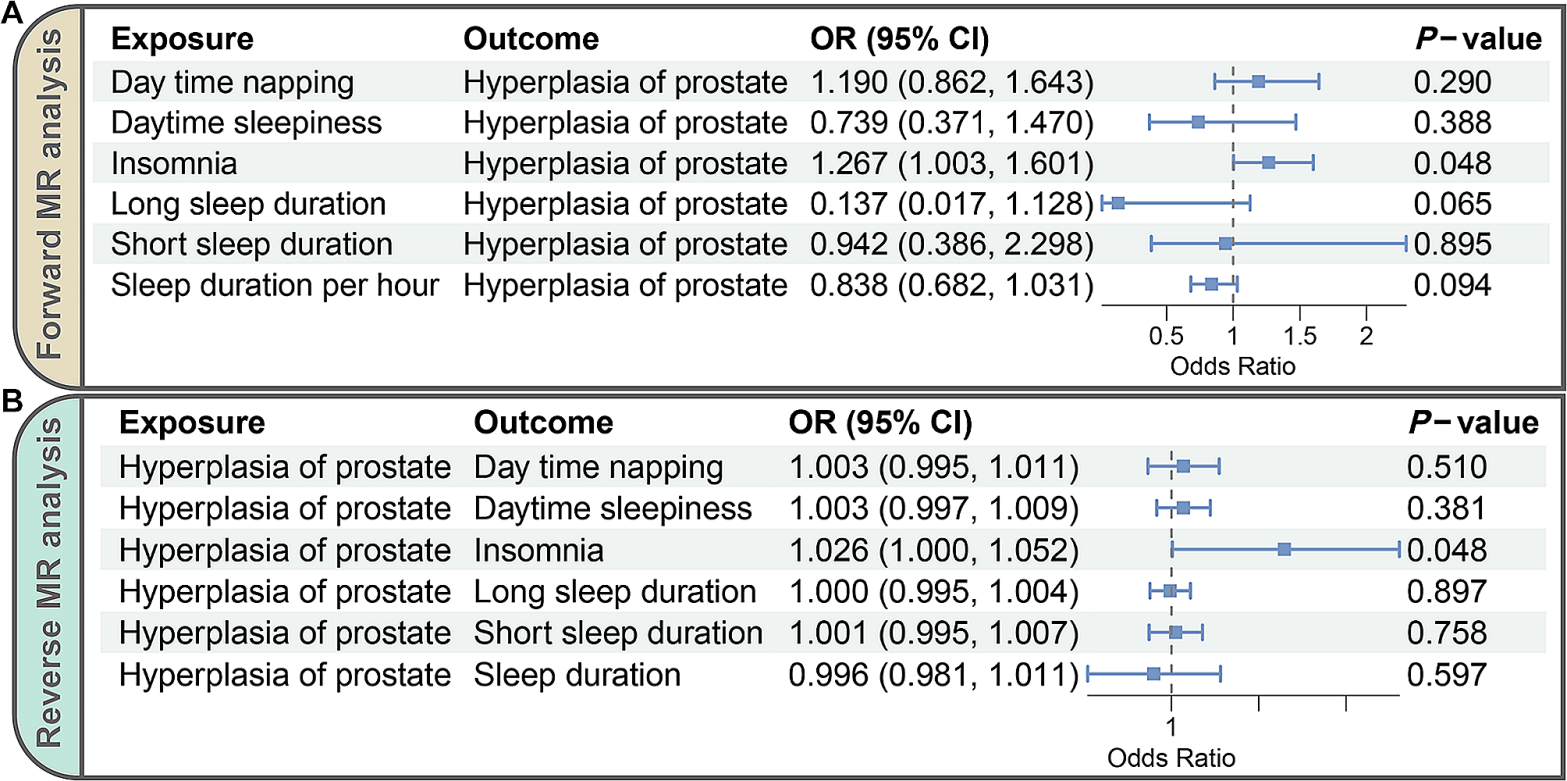
MR results identified by the IVW approach. (**A**) Forward MR analysis by IVW to assess the causal effects of six sleep traits on BPH. (**B**) Reverse MR analysis by IVW to assess the causal effects of BPH on six sleep traits

In forward MR analysis, IVW approach indicated that genetically predicted insomnia symptom elevated BPH risk (odds ratio [OR] = 1.267, 95% confidence interval [CI]: 1.003–1.601, *P* = 0.048) (Fig. 2A). However, genetically predicted day time napping (OR = 1.190, 95% CI: 0.862–1.643, *P* = 0.290), daytime sleepiness (OR = 0.739, 95% CI: 0.371–1.470, *P* = 0.388), long sleep (OR = 0.137, 95% CI: 0.017–1.128, *P* = 0.065), short sleep (OR = 0.942, 95% CI: 0.386–2.298, *P* = 0.895), and sleep duration per hour (OR = 0.838, 95% CI: 0.682–1.031, *P* = 0.094) did not affect BPH (Fig. 2A). Subsequently, the IVW approach in reverse MR analysis indicated that genetically predicted liability to BPH elevated the incidence of insomnia (OR = 1.026, 95% CI: 1.000-1.052, *P* = 0.048) (Fig. 2B). Nevertheless, BPH did not causally impact the other five sleep traits (*P* > 0.05) (Fig. 2B).

### Sensitivity tests verified the reliability of MR results

**Fig. 3:**
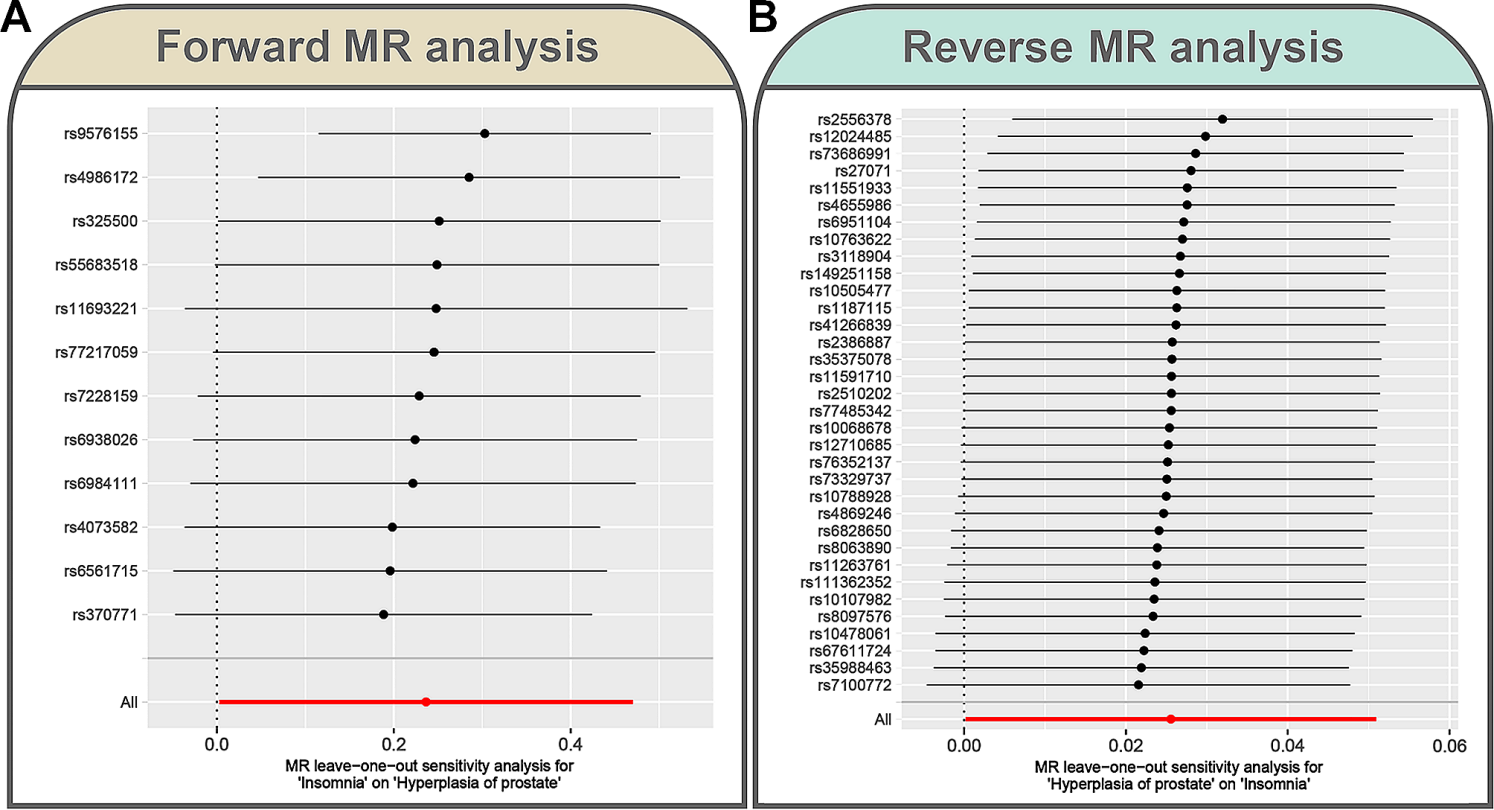
Assessment of the stability of MR results by leave-one-out sensitivity test. (**A**) MR leave − one − out sensitivity analysis for insomnia on BPH in forward MR analysis. (**B**) MR leave − one − out sensitivity analysis for BPH on insomnia in reverse MR analysis

The bidirectional causal association between insomnia and BPH was further validated by various sensitivity tests. First, four additional MR methods all showed consistent findings with IVW (OR > 1) (Table 1). Despite Cochran’s Q test indicating heterogeneity in the forward MR analysis (*P* < 0.05), it did not impact the MR inference as this study primarily employed the IVW random effects model (Table 2). In contrast, Cochran’s Q test indicated no heterogeneity in reverse MR analysis (*P* > 0.05) (Table 2). Subsequently, both the MR-Egger intercept test and the MR-PRESSO global test showed that the causal inference between insomnia and BPH was not significantly influenced by horizontal pleiotropy (*P* > 0.05) (Table 3). Ultimately, leave-one-out test demonstrates the stability, as excluding each IV does not lead to significant alterations in the results (Fig. 3).

**Table 1:** Identification of bidirectional causal relationship between insomnia and BPH by six distinct MR methods

Exposure	Outcome	MR method	OR (95% CI)	*P*-value
Insomnia	Hyperplasia of prostate	IVW	1.267 (1.003, 1.601)	0.048
		MR Egger	1.296 (0.678, 2.478)	0.451
		Weighted median	1.283 (0.995, 1.655)	0.055
		Maximum likelihood	1.281 (1.073, 1.530)	0.006
		Weighted mode	1.257 (0.890, 1.777)	0.221
Hyperplasia of prostate	Insomnia	IVW	1.026 (1.000, 1.052)	0.048
		MR Egger	1.015 (0.930, 1.109)	0.739
		Weighted median	1.023 (0.987, 1.061)	0.213
		Maximum likelihood	1.027 (1.001, 1.053)	0.046
		Weighted mode	1.028 (0.961, 1.099)	0.424

**Table 2:** Assessment of heterogeneity by Cochran’s Q test

Exposure	Outcome	Method	Cochran’s Q test		
			Q	Q_df	Q_pval
Insomnia	Hyperplasia of prostate	IVW	20.202	11	0.043
		MR Egger	20.191	10	0.028
Hyperplasia of prostate	Insomnia	IVW	30.697	33	0.582
		MR Egger	30.638	32	0.535

**Table 3:** Assessment of horizontal pleiotropy by MR-Egger intercept test and MR-PRESSO global test

Exposure	Outcome	MR-Egger intercept test			MR-PRESSO global test	
		Intercept	SE	*P* - value	RSS obs	*P* - value
Insomnia	Hyperplasia of prostate	-9.57E-04	0.013	0.942	23.472	0.059
Hyperplasia of prostate	Insomnia	9.51E-04	0.004	0.810	32.620	0.591

### Results of replicate MR analysis

The IVs screening process for the reverse MR analysis is shown in Supplementary Table S8. Ultimately, 75 IVs proxying for insomnia were obtained for the replicate MR analysis (Supplementary Table S9). In the replicate MR analysis based on male participants, IVW approach showed that genetically predicted insomnia symptom significantly increased the risk of BPH (OR = 1.488, 95% CI: 1.096–2.022, *P* = 0.011), which was more remarkable than the effect in the initial forward analysis (Supplementary Table S10). In addition, the remaining four MR. The results of the other four supplementary MR methods showed the same directionality as IVW. In addition, Cochran’s Q test showed that the replicate analysis was not affected by significant heterogeneity (*P* > 0.05) (Supplementary Table S11), whereas the MR-Egger intercept test and MR-PRESSO global test showed that the replicate analysis was not affected by significant levels of horizontal pleiotropy (*P* > 0.05) (Supplementary Table S12). The leave-one-out test indicated the stability of the results (Supplementary Fig. S1).

## Discussion

This MR study comprehensively assessed the causal associations between six sleep traits and BPH. Ultimately, a bidirectional positive causal relationship between genetically predicted insomnia symptom and liability to BPH was identified. Furthermore, various sensitivity analyses validated the reliability of the results.

Several observational investigations suggested an association between sleep traits and benign prostatic hyperplasia. An investigation involving middle-aged and elderly males in the Longitudinal Aging Study in India found a notable correlation between diminished sleep quality and elevated occurrences of BPH [6]. A cross-sectional study derived from the West China Natural Population Cohort Study revealed a substantial link between inadequate sleep quality and a heightened BPH risk, particularly prevalent among older male individuals [7]. Another cross-sectional study indicated a noteworthy correlation between shortened sleep duration and heightened susceptibility to BPH among middle-aged and elderly Chinese men [28]. The present MR study showed that prolonged insomnia significantly raises the risk of BPH, and BPH similarly heightens the risk of insomnia, thus providing more robust evidence for previous observational studies. More importantly, this study emphasizes the importance of improving sleep quality to reduce insomnia, thereby preventing BPH, and breaking the vicious cycle of insomnia-BPH-insomnia.

The bidirectional positive causal association between insomnia and BPH identified in our study could be rationalized by several mechanisms. A principal mechanism might be its influence on the regulation of hormones, particularly androgens such as testosterone [29]. The fragmentation of sleep seen with advancing age interrupts the circadian rhythm governing hormone release, including that of testosterone, which is known to have a significant role in BPH pathogenesis [29]. Studies indicate that nocturnal sleep time serves as a predictor for morning testosterone levels [30], underscoring the essential role of sufficient and uninterrupted sleep in maintaining hormone equilibrium. Furthermore, insomnia may result in the dysregulation of autonomic nervous system activity [31], a recognized pivotal factor in the progression of BPH [32]. Furthermore, insomnia might trigger elevate systemic inflammation [33], a condition implicated in the progression of BPH [34]. Moreover, sleep deficits are implicated in metabolic dysregulation, such as dysglycemia and insulin resistance [35], which, in turn, heightens the risk for BPH [36]. Another potential mechanism could be caused by depression, as previous studies have shown a bidirectional association of depression with both BPH and insomnia [37–39]. This complex mechanism may be further investigated in future multivariable MR analyses. Nevertheless, the pathways connecting insomnia and BPH have not yet been fully understood and warrant further investigations.

The current research exhibits several advantages. First, using MR analyses, the effects of varying lifetime levels of the six sleep traits on BPH were simulated, thus modeling long-term RCTs of sleep interventions. Second, the summary statistics of exposures and outcomes were derived from two distinct cohorts, reducing the high false-positive rate triggered by overlapping samples. Third, MR analysis was performed by screening IVs based on three MR core assumptions, and various sensitivity tests were implemented to increase the reliability of the results.

This study has several limitations that need to be clarified. First, this study was performed using GWAS summary statistics derived from European individuals. Consequently, the generalizability of the findings to other populations remains uncertain. Second, the data on sleep traits in the previous studies were obtained from questionnaires and might be influenced by recall bias. Third, the findings of this study solely provided suggestive correlational evidence due to lack of multiple testing correction, underscoring the necessity for future validation in larger cohorts. Finally, due to the inaccessibility of individual-level statistics, only summary data stratified by gender were available for replicate analysis, while analysis based on other clinical characteristics such as age was not feasible.

## Conclusions

Overall, through bidirectional two-sample MR analysis, this study identified that a higher prevalence of genetically predicted insomnia symptoms may significantly increase the risk of BPH, while liability to BPH may in turn increases the incidence of insomnia. Therefore, improving sleep quality and reducing the risk of insomnia could be a crucial approach for the prevention of BPH.

### Electronic supplementary material

Below is the link to the electronic supplementary material.

Supplementary Material 1

Supplementary Material 2

## Data Availability

Source of summary statistics is shown in the Supplementary Material.

## Abbreviations

BPH: Benign prostatic hyperplasia
MR: Mendelian randomization
IVs: instrumental variables
GWAS: genome-wide association study
RCTs: randomized controlled trials
SNPs: single-nucleotide polymorphisms
IVW: inverse variance weighed
OR: odds ratio
CI: confidence interval
