# Supplementary figures and images for: Mendelian randomization study supports positive bidirectional causal relationships between genetically predicted insomnia symptom and liability to benign prostatic hyperplasia
Source: BMC Urol. 2024 Apr 20;24:91. doi: 10.1186/s12894-024-01474-z (PMC11031934; doi:10.1186/s12894-024-01474-z)

Supplementary Figure S1. leave-one-out sensitivity test for replicate MR analysis

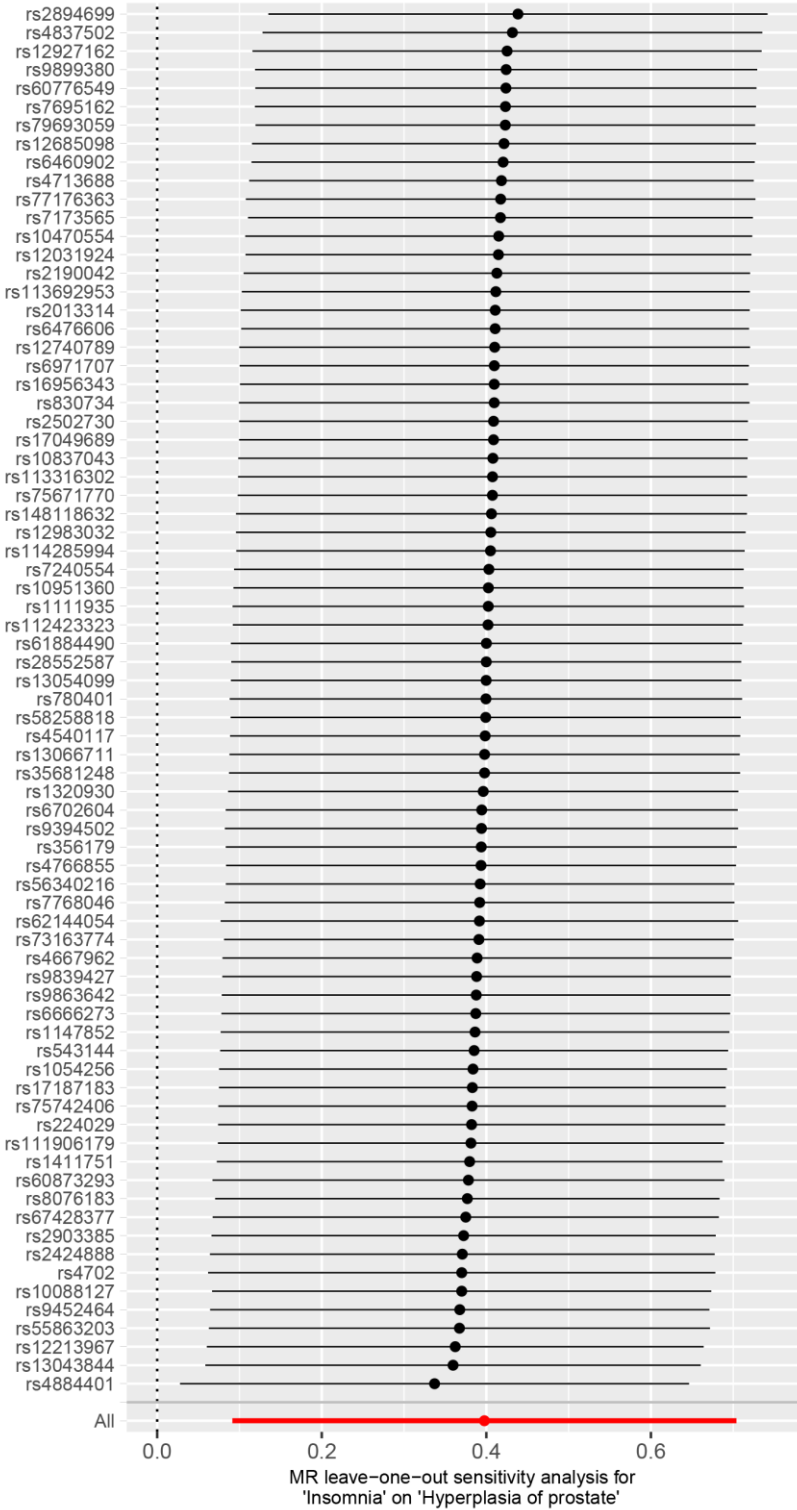

Supplement: Supplementary file 2 — Supplementary Material 2 [file 12894_2024_1474_MOESM2_ESM.pdf]
